# Pharmacological inhibition of protein tyrosine phosphatase 1B protects against atherosclerotic plaque formation in the LDLR^−/−^ mouse model of atherosclerosis

**DOI:** 10.1042/CS20171066

**Published:** 2017-09-29

**Authors:** Dawn Thompson, Nicola Morrice, Louise Grant, Samantha Le Sommer, Emma K. Lees, Nimesh Mody, Heather M. Wilson, Mirela Delibegovic

**Affiliations:** School of Medicine, Medical Sciences and Nutrition, University of Aberdeen, Aberdeen, U.K.

**Keywords:** atherosclerosis, metabolic syndromes, protein tyrosine phosphatases

## Abstract

Cardiovascular disease (CVD) is the most prevalent cause of mortality among patients with type 1 or type 2 diabetes, due to accelerated atherosclerosis. Recent evidence suggests a strong link between atherosclerosis and insulin resistance, due to impaired insulin receptor (IR) signalling. Here, we demonstrate that inhibiting the activity of protein tyrosine phosphatase 1B (PTP1B), the major negative regulator of the IR prevents and reverses atherosclerotic plaque formation in an LDLR^−/−^ mouse model of atherosclerosis. Acute (single dose) or chronic PTP1B inhibitor (trodusquemine) treatment of LDLR^−/−^ mice decreased weight gain and adiposity, improved glucose homeostasis and attenuated atherosclerotic plaque formation. This was accompanied by a reduction in both, circulating total cholesterol and triglycerides, a decrease in aortic monocyte chemoattractant protein-1 (MCP-1) expression levels and hyperphosphorylation of aortic Akt/PKB and AMPKα. Our findings are the first to demonstrate that PTP1B inhibitors could be used in prevention and reversal of atherosclerosis development and reduction in CVD risk.

## Introduction

Cardiovascular disease (CVD) is a general term used to describe all the conditions affecting the heart and blood vessels and is responsible for almost a third of deaths worldwide (WHO Statistics, http://www.who.int/mediacentre/factsheets/fs317/en/). Many conditions that contribute to CVDs are due to narrowing and hardening of the blood vessels through a process known as atherosclerosis, arising due to lipid accumulation which, over time, develops into plaques. Subsequently, these atherosclerotic plaques can lead to ischaemic injury by a number of mechanisms such as complete occlusion of the blood vessel or alternatively, the plaque may become unstable and rupture resulting in thrombosis [1,2]. This process may be exacerbated by risk factors encompassing genetic aspects, lifestyle choices such as smoking, excessive drinking, physical inactivity and obesity or conditions such as diabetes [3]. Indeed, in both type 1 and type 2 diabetic patients, a high proportion of mortality is associated with CVD, where defective insulin signalling leads to endothelial dysfunction and accelerated atherosclerosis. The mechanism contributing to this pathology is somewhat unclear; however, it has been suggested that insulin resistance and hyperglycaemia results in intracellular metabolic changes leading to oxidative stress and chronic low-grade inflammation [4]. Therefore, clarification of the mechanism controlling these diseases is needed to enable the design of more targeted and effective therapeutics.

In support of a link between defective insulin receptor (IR) signalling and atherogenesis, it was found that apolipoprotein-E-deficient mice (ApoE^−/−^) devoid of IR in the vascular endothelium had increased plaque development [5]. Moreover, ApoE^−/−^ mice with a heterozygous deletion of the IR and its downstream target, IR substrate 1 (IRS1), also develop accelerated atherosclerosis [6], as well as the mice lacking IR substrate 2 (IRS2^−/−^) [7]. Furthermore, decreased insulin signalling in nonhaematopoietic cells, as achieved by transplantation of ApoE^−/−^ mouse model of atherosclerosis with bone marrow cells from IRS1^+/−^ IR^+/−^ ApoE^−/−^ mice, contributed to increased atherogenesis in these mice [6]. Finally, mice that were devoid of both LDLR^−/−^ and Akt2, an important downstream component of the IR signalling cascade, exhibited impaired glucose homoeostasis, elevated insulin and cholesterol levels and developed more complex atherosclerotic plaques [8]. Therefore, targeting components that inhibit IR signalling could prove to be an effective therapeutic.

Protein tyrosine phosphatase (PTP)1B (PTP1B) has been identified as the major negative regulator of the IR itself [9]. In mice, whole body PTP1B^−/−^ studies established PTP1B as a major regulator of insulin sensitivity and body mass, via inhibition of insulin and leptin signalling respectively [10,11]. Our recent data suggested that hepatic-specific deletion of PTP1B, in addition to improving glucose and lipid homoeostasis and increasing insulin sensitivity, was protective against endothelial dysfunction in response to high fat diet (HFD) [12]. This was also associated with decreased hepatic inflammation in these mice [13]. Specifically, mice lacking hepatic PTP1B exhibited decreased systolic and diastolic blood pressure, in response to HFD feeding, when compared with control littermates [12]. Furthermore, we have also shown that myeloid-specific PTP1B deletion can protect against HFD-induced inflammation and hyperinsulinaemia and is facilitated by an increase in the secretion of the anti-inflammatory cytokine interleukin (IL)-10 (IL-10) and a decrease in pro-inflammatory TNFα cytokine secretion [14]. Since atherosclerosis is regarded as a chronic low level inflammatory disease [15–18], we hypothesized that targeting PTP1B activity using a PTP1B-specific inhibitor trodusquemine [19], could prove effective in prevention and possibly reversal of atherosclerotic plaque formation. This would enable direct testing of the translational potential of PTP1B inhibitors [20], which are in phase II clinical trials for diabetes treatment, and phase I clinical trials for breast cancer treatment (https://clinicaltrials.gov/ct2/show/NCT02524951), as treatments for atherosclerosis and reduction in CVD risk. To directly test this, we used the LDLR^−/−^ mouse model of atherosclerosis, under physiological and obesogenic conditions.

## Methods

### Animal studies

All animal procedures were performed under a project license approved by the U.K. Home Office under the Animals (Scientific Procedures) Act 1986 (PPL 60/3951). Eight weeks old male LDLR^−/−^ mice were purchased from Jackson Labs, individually housed and maintained at 22–24°C on 12-h light/dark cycle with free access to food/water. Following 2 weeks of acclimatization time, mice were placed on chow or HFD (42% from fat, 0.2% cholesterol, Envigo, Huntingdon, U.K.) for 12 weeks and weighed weekly to monitor weight gain.

### Drug treatments

The PTP1B inhibitor trodusquemine was obtained from Dr N. Tonks (Cold Spring Harbor, U.S.A.). After 1 week on HFD, 20 mice were injected intraperitoneally (I.P.) with the PTP1B inhibitor trodusquemine (10 mg/kg), followed by four subsequent weekly injections of 5 mg/kg, as previously described for *ob/ob* mice [19] and a 6-week washout period. These were designated as the chronic group, whereas the remaining mice were injected with saline. After 8 weeks on HFD, a further 20 mice were injected with a single dose of 10 mg/kg trodusquemine and designated accordingly, followed by a 4-week washout period. At week 12 on HFD, mice were fasted for 5 h and injected with either saline or insulin (10 mU/g body weight) for 10 min prior to culling by CO_2_ inhalation and subsequent cervical dislocation. Trodusquemine treatment was halted prior to the end of the study to ensure that the procedure of treatment (by intraperitoneal injection) did not affect the terminal signalling experiment by altering stress hormone levels and thus adversely affecting insulin signalling. Heart and aortic tissues were harvested and collected for further analysis. Tissues for subsequent Western blotting or qPCR analysis were frozen in liquid nitrogen and stored at –80°C until needed, whereas tissues for histology were immersed in formalin for 24 h at 4°C, then stored at 4°C in PBS until analysed.

### Glucose tolerance tests

Mice were fasted for 5 h prior to commencement of glucose tolerance tests (GTTs). Briefly, baseline glucose levels were sampled from tail blood using glucose meters (AlphaTRAK, Abbott Laboratories, Abbott Park, IL, U.S.A.). Subsequently mice were injected I.P. with 20% glucose (w/v) and blood glucose measured at 15, 30, 60 and 90 min post-injection.

### Body fat mass composition

The body composition of each mouse was analysed using an Echo MRI-3-in-1 scanner (Echo Medical Systems, Houston, TX, U.S.A.).

### Immunoblotting

Frozen aorta tissues were homogenized in 300 µl of ice-cold radioimmunoprecipitation assay (RIPA) buffer (10 mM Tris/HCl pH 7.4, 150 mM NaCl, 5 mM EDTA pH 8.0, 1 mM NaF, 0.1% SDS, 1% Triton X-100, 1% sodium deoxycholate with freshly added 1 mM NaVO_4_ and protease inhibitors) using a PowerGen 125 homogenizer and lysates normalized to 1 µg per 1 µl. Proteins were separated on a 4–12% bis-tris gel by SDS/PAGE and transferred on to nitrocellulose membrane. Membranes were probed for the following targets; p-IR (Tyr^1162/1163^), IR β-chain, p-AKT (Ser^473^), p-p38 (The^181^/Tyr^182^), total p-38, p-S6 (Ser^235/236^), total S6, p-AMPKα (Thr^172^), total AMPKα, PTP1B and GAPDH.

### RNA extraction and qPCR

Frozen tissues were lysed in TRIzol reagent (Sigma, U.K.) and RNA isolated using phenol/chloroform extraction according to manufacturer’s instructions. RNA was then synthesized into cDNA using tetrokits (Bioline) and subjected to qPCR analysis using SYBR and LightCycler 480 (Roche). Gene expression of intracellular cell adhesion molecule-1 (*ICAM-1*), vascular cell adhesion molecule-1 (*VCAM-1*) and monocyte chemoattractant protein-1 (*MCP-1*) was determined relative to the reference gene *ELF1*.

### Histology

Immediately following cervical dislocation, hearts were immersed in formalin and stored at 4°C for 24 h, before being transferred to PBS until further analysis. Hearts were bisected to remove the lower ventricles, frozen in OCT and subsequently sectioned at 5-μm intervals until the aortic sinus was reached. Sections were mounted and stained with Oil Red O to assess plaque formation. Images were captured using a light microscope and plaque formation quantified using ImageJ software.

### Serum analysis

Blood was collected during terminal procedures after fasting (5 h) and spun at 5000×***g*** to isolate serum, then stored at –80°C. Serum samples were subsequently analysed for insulin using ELISA (R&D Systems) or total cholesterol and triglycerides (Sigma).

### Statistical analysis

We expressed all the values as mean ± S.E.M. We determined group sizes by performing a power calculation to lead to 80% chance of detecting a significant difference (*P*≤0.05). For both *in vivo* and *ex vivo* data, each *n* value corresponds to a single mouse. Statistical analyses were performed by using one- or two-way ANOVA, followed by Tukey’s or Dunnett’s multiple-comparison tests to compare the means of three or more groups or by an unpaired two-tailed Student’s *t*test to compare the means of two groups. Variances were similar between groups. In all the figures, */^#^*P*≤0.05, **/^##^*P*≤0.01, ***/^###^*P*≤0.001, ****P≤0.0001. All analyses was performed using GraphPad Prism (GraphPad Software).

## Results

### PTP1B inhibitor treatment decreases body weight and improves global glucose homeostasis

The exponential rise in patients presenting with obesity and type 2 diabetes has resulted in an increased interest from pharmaceutical companies for the use of PTP1B inhibitors as potential therapeutics [20]. Given there is increasing evidence implicating defective insulin signalling as a major contributor to the pathogenesis of atherosclerosis, we hypothesized that PTP1B inhibition should have beneficial protective effects. To determine if global PTP1B inhibitors would attenuate plaque formation, we used the LDLR^−/−^ mouse model of atherosclerosis [21]. We assessed whether a single dose and/or chronic PTP1B inhibition could slow or reverse atherosclerotic plaque formation in mice fed on HFD (or chow as control). The PTP1B inhibitor trodusquemine was selected as this drug has been reported to be more specific than previously synthesized compounds, since it binds and inhibits allosterically rather than at the catalytic domain which is highly conserved between other tyrosine phosphatase family members [22]. Furthermore, this drug is currently in phase I trials in breast cancer patients (https://clinicaltrials.gov/ct2/show/NCT02524951), after previously being tested as a treatment for type 2 diabetes and obesity.

**Figure 1:**
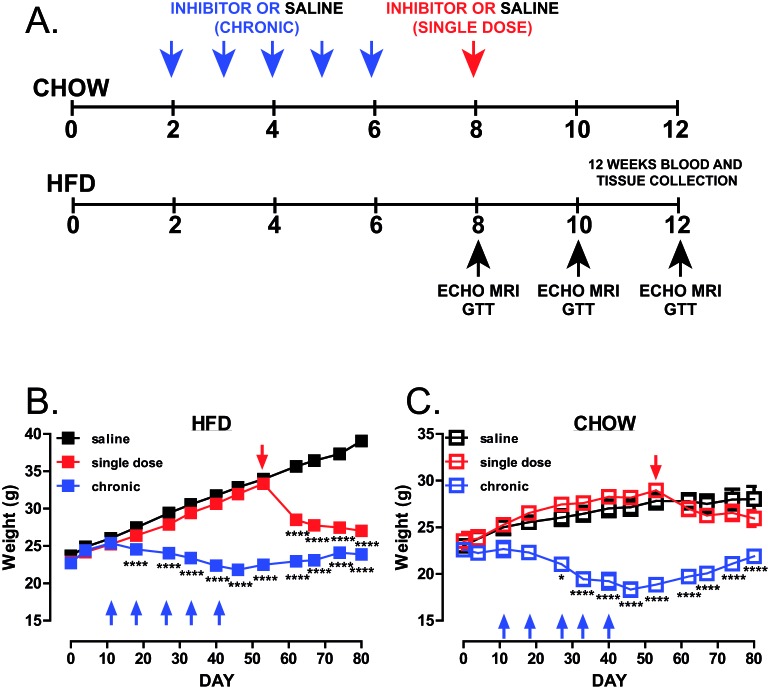
Global PTP1B inhibition leads to reduced body weight (**A**) Schematic representation of the experimental design. LDLR^−/−^ male mice were divided into HFD-fed and chow-fed saline-treated, single-dose trodusquemine (10 mg/kg I.P. at week 8) and chronic trodusquemine (a single 10 mg/kg I.P. followed by four weekly injections at 5 mg/kg). (**B**,**C**) Weights of mice during the course of the experiment-fed HFD (B, *n*=24 per group) or chow (C, *n*=4 per group). Data are represented as mean ± S.E.M. and analysed by two-way ANOVA followed by Bonferonni multiple comparison *t* tests where *P≤0.05, or ****P≤0.0001 when compared with saline-treated control mice.

**Figure 2:**
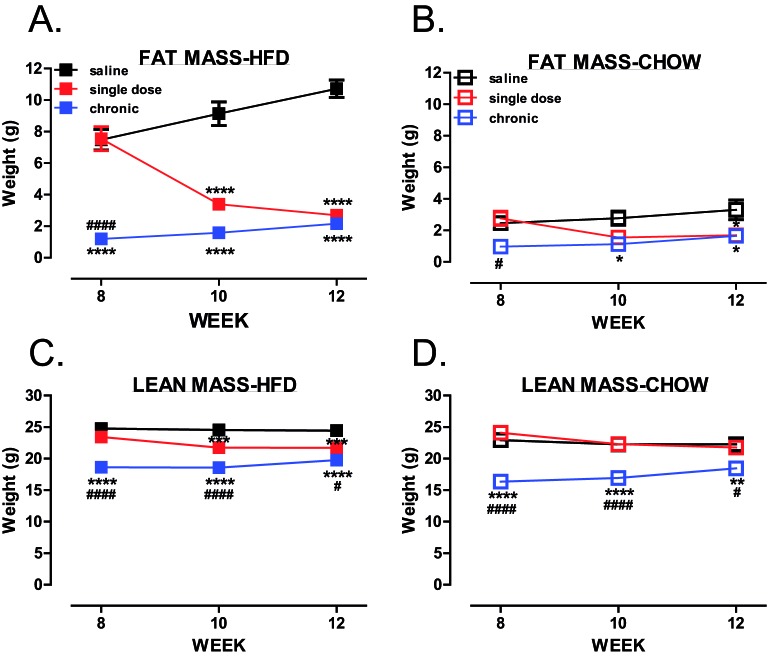
Global PTP1B inhibition leads to reduced adiposity (**A**–**D**) Body composition was analysed using an Echo MRI 3-in-1 scanner where total body fat (**A,B**) and lean mass (**C,D**) were determined (HFD, *n*=9–11 per group; chow, *n*=4 per group). Data are represented as mean ± S.E.M. and analysed by two-way ANOVA followed by Bonferonni multiple comparison *t*tests where *P≤0.05, **P≤0.01, ***P≤0.001, ****P≤0.0001 when compared with saline treated control mice or ^#^*P*≤0.05, ^####^*P*≤0.0001 when single dose and chronic groups were compared with each other.

**Figure 3:**
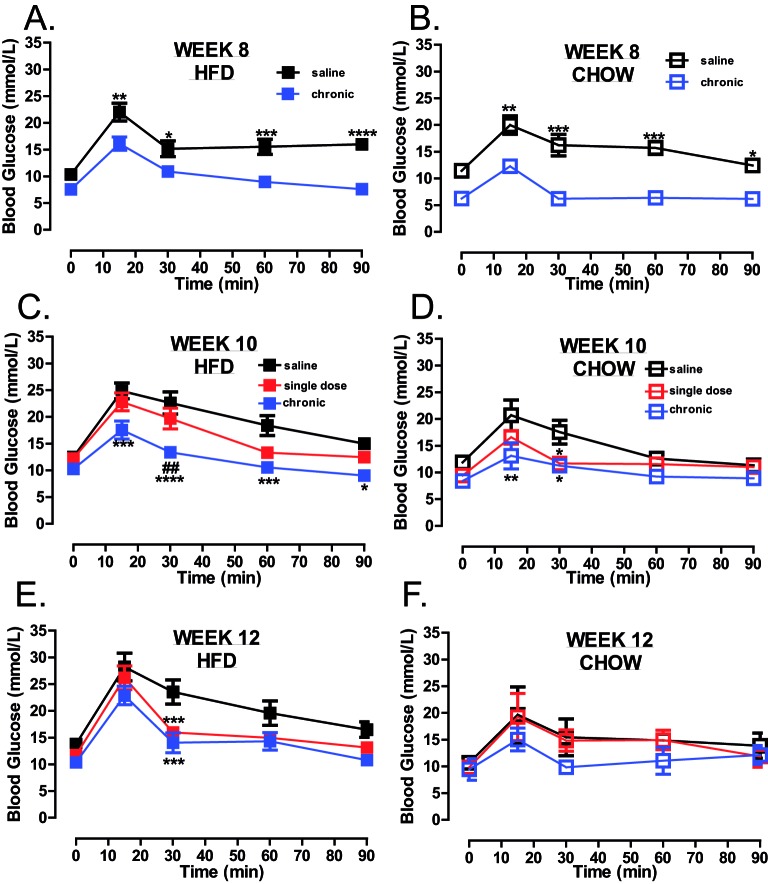
Global PTP1B inhibition improves glucose maintenance GTTs of saline, single dose and chronic drug treated mice fed on HFD (**A**,**C**,**E**) or chow (**B**,**D**,**F**) diets at week 8, 10 and 12. Mice were fasted for 5 h prior to basal glucose monitoring (as described in Methods section) and subsequently mice were injected I.P. with 20% glucose (w/v), and blood re-analysed at 15, 30, 60 and 90 min post-injection (HFD, *n*=8 per group; chow, *n*=4 per group). Data are represented as mean ± S.E.M. and analysed by two-way ANOVA followed by Bonferonni multiple comparison *t*tests where *P≤0.05, **P≤0.01, ***P≤0.001, ****P≤0.0001 when compared with saline treated control mice or ^##^*P*≤0.01 when single dose and chronic groups were compared with each other.

Mice were treated chronically with trodusquemine or given a single dose after 8 weeks of HFD feeding (Figure 1A). Similar to whole-body PTP1B deletion [10] and treatment of ob/ob mice using these inhibitors [19], chronic global inhibition of PTP1B prevented weight gain in both, chow and HFD-fed mice, when compared with saline controls (Figure 1B,C), and led to decreased fat mass (Figure 2A,B). Lean mass was significantly decreased after 6 weeks of treatment in chronically treated mice (Figure 2C,D), when compared with vehicle controls, but remained stable over 8–10 weeks, with no significant reduction in muscle mass when compared with vehicle-treated animals. Following 8 weeks on HFD, a single dose of trodusquemine led to a 20% reduction in body weight (Figure 1B), with greater than 50% reduction in fat mass, that accounted for the majority of the effect in weight loss (Figure 2A). In addition, in agreement to what has been previously shown [19], trodusquemine exposure led to reduced food intake in both HFD-fed and CHOW-fed cohorts (Supplementary Figure S1A,B). This was evidenced at week 9 which was 1 week following commencement of the single dose cohort. Interestingly, even though the chronic group had ceased drug treatment after week 6, a significant reduction in food intake was still present. However, PTP1B activity assays performed on liver tissues collected at week 12 during terminal culls revealed no significant inhibition of PTP1B remained in drug treated cohorts (Supplementary Figure S1C). The lack of inhibition observed is likely to be due to drug washout, since single dose and chronic cohorts had 4 and 6 weeks respectively, in the absence of inhibitor prior to culling. Finally, in both, chronic and single dose inhibitor treated mice, there was also a significantly improved glucose handling in HFD-fed mice at all time points (weeks 8, 10 and 12 (Figure 3A,C,E respectively) whereas, although improved in chow-fed mice at week 8 and 10 (Figure 3B,D), this was not evident at week 12 (Figure 3F).

**Figure 4:**
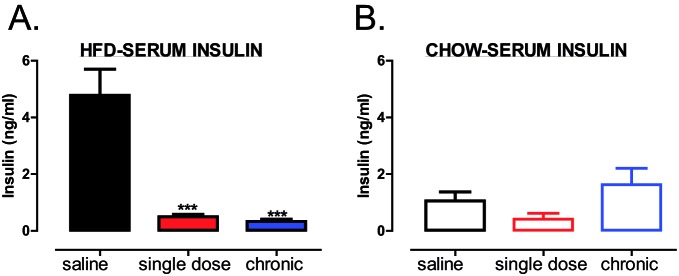
Global PTP1B inhibition reduces circulating insulin levels in HFD-fed mice (**A**,**B**) Serum from blood collected at terminal culls was analysed for circulating insulin levels in HFD-fed (**A**) and chow-fed (**B**) mice using ELISA (Millipore). Data are represented as mean ± S.E.M. and analysed by unpaired *t*tests where ***P≤0.001 when compared with saline treated control mice.

Previous research has shown that HFD-fed mice develop hyperinsulinaemia [23,24]. In agreement with these studies, HFD led to an increase in circulating insulin levels in saline treated mice (Figure 4A), whereas, a significant decrease in circulating insulin levels was observed in HFD-fed, but not in chow-fed mice, treated either with a single dose or chronically with trodusquemine (Figure 4A,B respectively). Therefore, global inhibition of PTP1B, using PTP1B specific inhibitor, mirrored results previously observed in whole-body PTP1B knockout mice, with regards to beneficial effects on body weight, adiposity and glucose homoeostasis maintenance [10,11].

### PTP1B inhibitor treatment protects against and reverses obesity-induced increase in atherosclerotic plaque area

**Figure 5:**
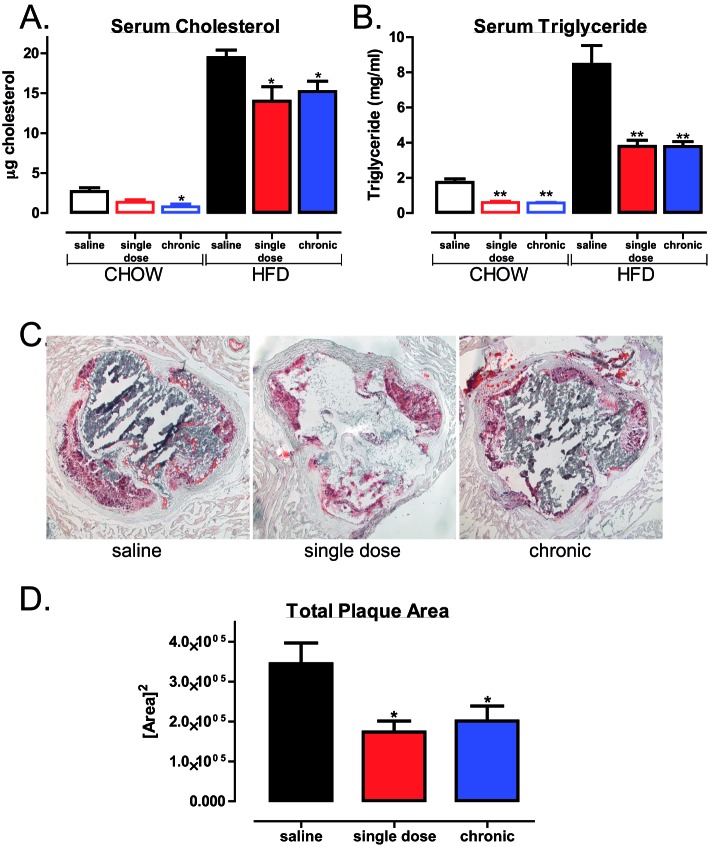
Global PTP1B inhibition reduces serum total cholesterol and triglycerides and prevents atherosclerotic plaque development Blood was collected at terminal culls and serum analysed for circulating total cholesterol (**A**) triglyceride levels (**B**) using ELISA (Sigma). (**C**) Representative (*n*=5–6 per group) aortic root sections of HFD-fed mice stained with Oil Red O. (**D**) Quantification of plaque area as analysed using ImageJ software. Data are represented as mean ± S.E.M. and analysed by unpaired two-tailed *t*tests where *P≤0.05 or **P≤0.01 when compared with saline control.

Increased blood lipid and lipoproteins are widely used as biomarkers to predict CVD risk [25]. To assess the lipid lowering potential of PTP1B inhibition, lipid analyses were performed. A single dose and chronic treatment with trodusquemine resulted in significantly decreased serum cholesterol (Figure 5A) and triglyceride levels (Figure 5B) in HFD mice. A similar decrease was also measured in chow-fed trodusquemine-treated mice (Figure 5A,B). Subsequently, both single dose and chronic trodusquemine treatment resulted in attenuated plaque formation, as indicated by a decrease in total plaque area (Figure 5C,D). Therefore, we show, for the first time, that use of global PTP1B inhibitor not only decreases weight gain and improves glucose maintenance, but also decreases and most importantly reverses atherosclerotic plaque formation in an LDLR^−/−^ mouse model of atherosclerosis, under obesogenic HFD-feeding conditions.

**Figure 6:**
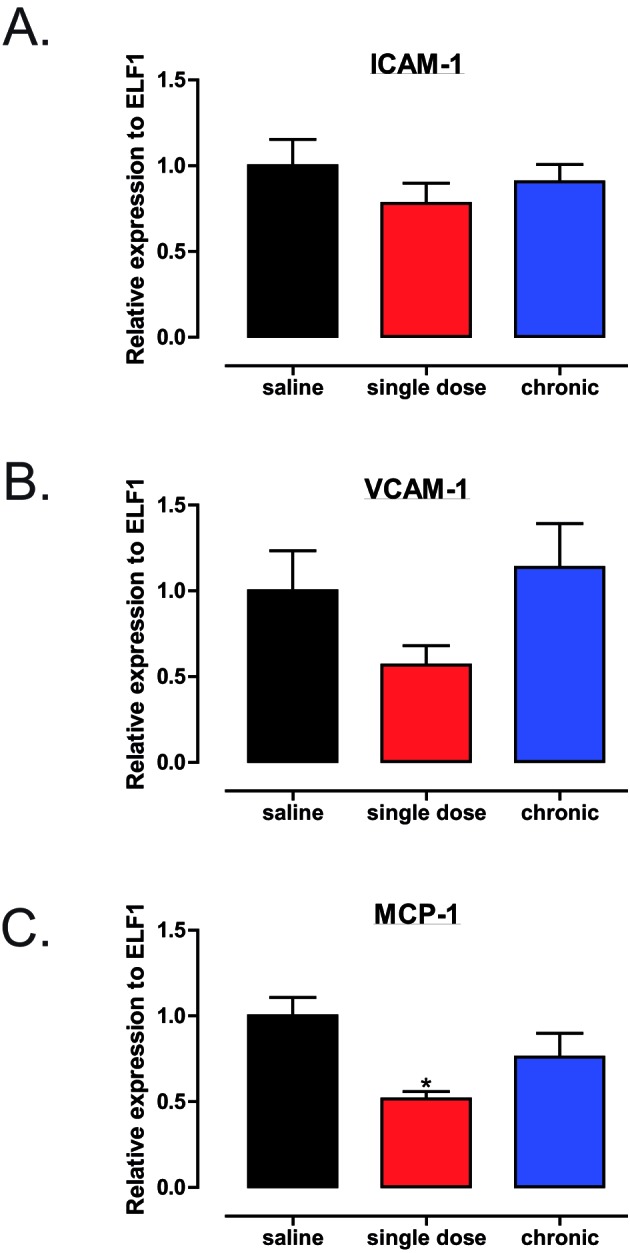
Single-dose global PTP1B inhibition reduces MCP-1 expression (**A**–**C**) Genetic analysis of aortic tissues (*n*=6 per group) as analysed by qPCR using using SYBR and Light Cycler 480 (Roche). Gene expression of *ICAM-1, VCAM-1* and *MCP-1* was determined relative to the reference gene *ELF1*. Data are represented as mean ± S.E.M. and analysed by unpaired two-tailed *t* tests where *P≤0.05 when compared with saline control.

Atherosclerosis is now widely regarded as a chronic, low-grade inflammatory condition characterized by an increased pro-inflammatory environment and decreased anti-inflammation, pro-resolutionary signalling [26,27]. Thus, a vicious cycle ensues and a failure of the tissue to return to homeostasis. Therefore, we investigated the expression of genes important in the inflammatory response including MCP-1, ICAM-1 and VCAM-1. MCP-1 is responsible for recruiting monocytes to the aortic tissue whereas both ICAM-1 and VCAM-1 enable their transmigration [7]. Although there were no changes in the expression of aortic ICAM-1 (Figure 6A) or VCAM-1 (Figure 6B), in either chronic or saline treated mice, those animals treated with a single injection of trodusquemine exhibited attenuated aortic MCP-1 expression levels (Figure 6C). Hence, suggesting less monocyte recruitment and a reduced inflammatory environment which could contribute to the reduction in plaque development.

### Decrease in atherosclerotic plaque area with PTP1B inhibitor treatment is accompanied by hyperphosphorylation of aortic Akt and AMPKα1

**Figure 7:**
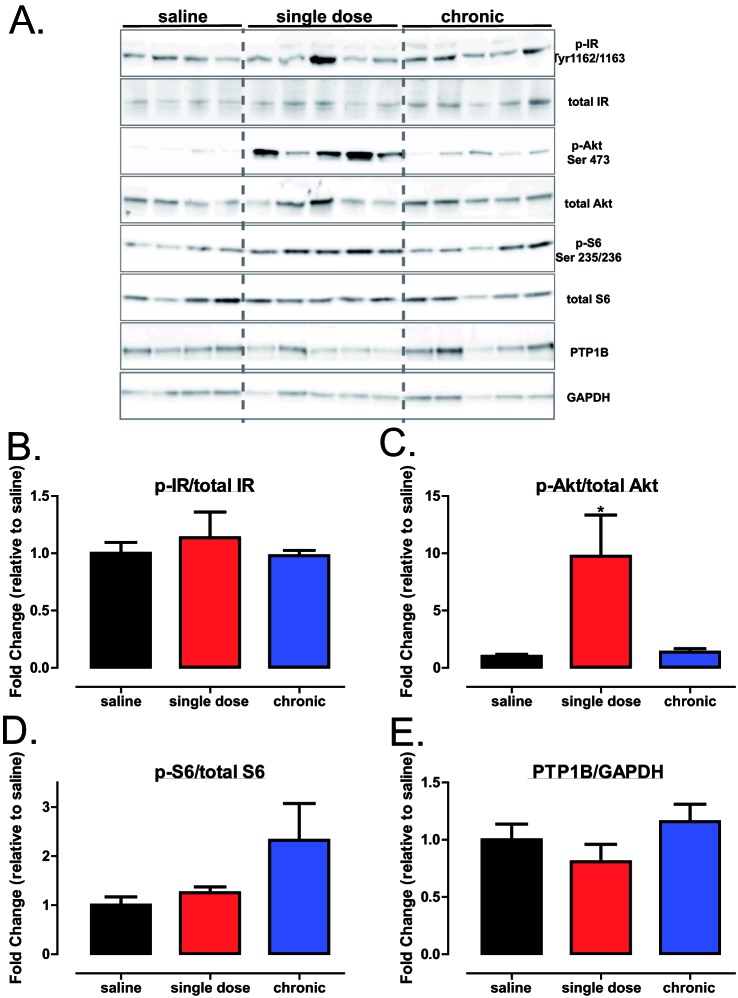
Global PTP1B inhibition improves aortic Akt signalling (**A**) Western blot analysis of aortic tissues from saline, single dose and chronic HFD cohorts injected with insulin immediately prior to culling. Quantification of p-IR (Tyr^1162/1163^) (**B**), p-Akt (Ser^473^) (**C**), p-S6 (Ser^235/236^) (**D**) and total PTP1B (**E**) using ImageJ software. Data are represented as mean ± S.E.M. and analysed by one-way ANOVA followed by Dunnett’s *t* tests where *P≤0.05 when compared with saline control.

**Figure 8:**
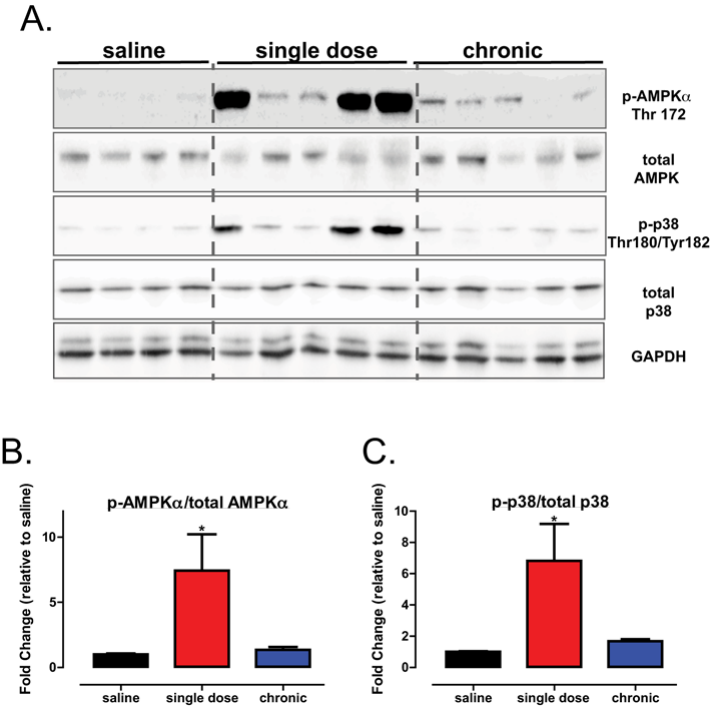
Global PTP1B inhibition improves aortic AMPK signalling **(A**) Western blot analysis of aortic tissues from saline, single dose and chronic HFD cohorts injected with insulin immediately prior to culling. Quantification, p-AMPKα (Thr^172^) (**B**) and p-p38 (Thr^180^/Tyr^182^) (**C**) using ImageJ software. Data are represented as mean ± S.E.M. and analysed by one-way ANOVA followed by Dunnett’s *t*tests where *P≤0.05 when compared with saline control.

Given the decrease in serum lipids and aortic plaque formation in trodusquemine-treated mice, we hypothesized that an up-regulation of IR signalling and associated downstream pathways could account for the beneficial effects of PTP1B inhibitors in our study. However, in contrast with our hypothesis, there was no significant increase in the aortic IR phosphorylation in either of our drug-treated mice cohorts (Figure 7A,B). However, Akt phosphorylation was significantly increased in the aortas from those mice receiving a single injection of trodusquemine (Figure 7A,C), without significant alterations in pS6 (Figure 7A,D). There were no differences in the expression levels of aortic PTP1B in trodusquemine-treated mice when compared with the saline controls (Figure 7E), as expected, as the inhibition of PTP1B with trodusquemine treatment has been shown to selectively inhibit PTP1B activity [19]. Interestingly, there was also a significant increase in the aortic AMPKα1 phosphorylation and downstream p38 (Figure 8A–C respectively) in single-dose trodusquemine treated mouse aortas. Finally, previous research from our lab has found deletion of hepatic PTP1B can improve the endoplasmic reticulum (ER) stress response [13,28], therefore, we sought to determine the effect of trodusquemine treatment on markers of ER stress. There was no significant improvement in either inositol-requiring enzyme 1α (IRE1α) or in binding of immunoglobulin protein (BiP) across treatments (see Supplemetary Figure S2A–C). However, a single dose of trodusquemine had opposing effects, leading to increased phosphorylation of eukaryotic translation initiation factor 2a (eif2a) but a significant decrease in the expression of C/EBP homologous protein (CHOP) (Supplementary Figure S2A,D,E). These data suggest that the beneficial effects of PTP1B inhibition cannot be attributed to direct regulation of the IR itself, but instead involves an Akt-AMPKα1-dependent mechanism.

## Discussion

We demonstrate here, using the LDLR^−/−^ mouse model of atherosclerosis, that pharmacological PTP1B systemic inhibition leads to protection against and reversal of atherosclerosis development, suggesting beneficial effects of PTP1B inhibition for the treatment of CVDs and reduction in CVD risk. We present evidence that, in addition to its improvement in glucose homeostasis and adiposity, PTP1B inhibition results in activation of aortic Akt and AMPKα1, that is independent of the effects on the IR itself. Most importantly, for the first time, we demonstrate that inhibition of PTP1B results in a decrease in circulating serum cholesterol and triglyceride levels and protection against atherosclerotic plaque formation.

Our findings complement our previous genetic research, where we demonstrated deletion of hepatic PTP1B protected against HFD-induced endothelial dysfunction, without altering body mass or adiposity [12]. However, in contrast with our original hypothesis, this was not a consequence of improvements in IR phosphorylation. Nonetheless, these data are in agreement with several other studies from our lab, specifically both the liver inducible [28] and the myeloid [14] PTP1B deletion models where, although exhibiting improved glucose homeostasis, did so independently of the IR signalling cascade, suggestive of multiple targets for the beneficial effects of PTP1B inhibition. Likewise, PTP1B deletion within adipocytes was unable to improve IR signalling within this tissue [29]. Therefore, this is suggestive that PTP1B inhibition, in addition to its anti-diabetic role, may also exert its actions through different mechanism(s).

In the past few years, in support of our findings, there have been several studies supporting a beneficial role for PTP1B in endothelial dysfunction that is independent of IR signalling but, instead, dependent on that of vascular endothelial growth factor (VEGF) signalling through the negative regulation of VEGF receptor 2 (VEGFR2) (see Thiebaut et al. [30] for a recent review). Pharmacological inhibition or genetic deletion of PTP1B improved heart failure due to the beneficial effects on cardiac remodelling, such as increased contractile function, and a decrease in cardiac hypertrophy in fibrosis [31]. A similar phenotype was observed in a model of sepsis, where whole-body PTP1B deletion not only improved survival rate in response to septic shock, but decreased cardiac dysfunction and the expression of pro-inflammatory markers such as IL1β, ICAM-1, VCAM-1, COX-2 and iNOS [32]. Furthermore, a follow-up study where PTP1B was specifically deleted in endothelial cells, demonstrated again cardiac improvement exhibiting increased survival after 20 weeks post-induction of heart failure [33]. Critically, these improvements were accompanied by an increase in VEGFR signalling and angiogenesis. Finally, in a model of hind limb ischaemia, deletion of PTP1B in endothelial cells led to angiogenesis and arteriogenesis both *in vitro* and *in vivo*, and was mediated by enhanced VEGFR signalling [34]. Therefore, the possibility that improved VEGFR signalling is involved in the beneficial effects observed in trodusquemine treated mice, although not investigated during the present study, cannot be ruled out and is worth future investigation. Likewise, the effect of trodusquemine treatment on additional cell types not limited to the vasculature, such as those involved in the immune response must also be considered, as trodusquemine acts as the global PTP1B inhibitor.

Nonetheless, importantly, we observed enhancement of aortic AMPK α1 phosphorylation in HFD-fed mice given a single dose of trodusquemine. This is in agreement with a similar recent study in which the PTP1B global knockout exhibited improved cardiomyocyte contractility in mice fed on HFD, through an AMPK-dependent mechanism [35]. In addition, an independent study using the LDLR^−/−^ mouse model of atherosclerosis, found deletion of AMPKα1 specifically in the myeloid lineage, led to hypercholesterolemia, increased macrophage inflammation and plaque infiltration and exacerbated atherogenesis [36]. Therefore, the robust phosphorylation of aortic AMPKα1 observed in response to a single injection, and to some extent, chronic global PTP1B inhibition with trodusquemine, and the associated protection and reversal of atherosclerotic plaque area, suggest that PTP1B inhibition may be protective through an AMPKα1-driven mechanism. It is important to note that at the time of culling, single dose and chronic drug treated mice had 4- and 6-weeks washout of drug respectively. This 2-week difference may explain why chronic treatment did not exhibit phosphorylation of AMPK α1 or Akt to the same extent as those given a single injection. A group of mice receiving single or chronic trodusquemine with no washout period would be required to assess if trodusquemine could directly lead to hyperphosphorylation of AMPKα1 and Akt. However, despite this, both drug treated cohorts exhibited the same degree of decreased plaque formation.

Critically, atherosclerosis is now well regarded as a chronic low-level inflammatory disease accompanied by a failure to initiate anti-inflammatory signalling, thereby preventing successful engagement of pro-resolution mechanisms and a return to tissue homeostasis. In contrast with previous therapies, including those which inhibit pro-inflammatory signalling, current research is focusing on the promotion from pro-inflammation to pro-resolution, as a means to reduce atherosclerotic plaque development, as well as other chronic inflammatory pathologies [37,38]. Our study revealed a decrease in MCP-1 expression in trodusquemine-treated mice, suggesting that PTP1B inhibition led to a less pro-inflammatory environment. Furthermore, in our model where PTP1B deletion was myeloid specific, these mice exhibited a decrease in pro-inflammatory IL-6 and TNFα, and an increase in pro-resolution IL-10 [14]. Finally, a recent study by Zhu et al. [39] found that IL-10 stimulation of AMPKα phosphorylation and subsequent downstream PI3K/Akt/mTORC1 signalling was critical for eliciting the anti-inflammatory properties of this cytokine. Therefore collectively, these data suggest that PTP1B inhibition may contribute in the switch from pro-inflammation to pro-resolution signalling, via IL-10/AMPKα mechanism.

In conclusion, we demonstrate that global pharmacological inhibition of PTP1B, in addition to its anti-diabetic and weight loss benefits, resulted in both the reduction and reversal in atherosclerotic plaque formation under obesogenic conditions (as achieved by chronic and a single dose exposure, respectively). This was achieved via an IR-independent pathway, and instead engaged Akt/AMPKα signalling to promote a decrease in pro-inflammatory environment. Hence our data strongly suggest that PTP1B inhibitors may be used in pathologies other than type 2 diabetes and that those currently in pre-clinical trials [20], could be repurposed to target chronic inflammatory pathologies, such as atherosclerosis and help to reduce CVD risk.

## Clinical perspectives

- CVD is the most prevalent cause of mortality among patients with type 1 or type 2 diabetes due to accelerated atherosclerosis.
- Inhibiting the activity of PTP1B prevents and reverses atherosclerotic plaque formation in LDLR^−/−^ mouse model of atherosclerosis, and is associated with a decrease in aortic MCP-1 expression levels, hyperphosphorylation of aortic Akt/PKB and AMPKα.
- Our findings are the first to demonstrate that PTP1B inhibitors could be used in prevention and reversal of atherosclerosis.

## Supporting information

**Supplemental Figure 1:** **Trodusquemine treatment reduces food intake.** Food intake of HFD (**A**) and CHOW-fed (**B**) cohorts at week 9. PTP1B activity data of liver tissue from HFD-fed saline, single dose or chronically trodusquemine treated cohorts following terminal culls as assessed by inhibition of free phosphate production (C, n=4-5 per group). Data are represented as mean ± S.E.M. and analysed by unpaired two tailed t-tests where *p≤0.05 or ****p≤0.0001 when compared to saline control groups.

**Supplemental Figure 2:** **Trodusquemine treatment does not improve ER stress.** (**A**) Western blot analysis of aortic tissues from saline, single dose or chronically treated HFD-fed mice injected with either saline or insulin immediately prior to culling. Quantification of p- pIRE1α (Ser 727) (**B**), BiP (**C**) p-eif2α (Ser 51) (**D**) and CHOP (**E**) represented in (**A**) Data are represented as mean ± S.E.M. and analysed by unpaired two-tailed t-tests.

## Abbreviations

ApoE^−/−^: apolipoprotein-E-deficient mice
AMPK: AMP-activated kinase
C/EBP: CCAAT/enhancer binding protein
COX-2: cyclooxegenase-2
CVD: cardiovascular disease
Elf1: E74-like factor 1
ER: endoplasmic reticulum
GAPDH: glyceraldehyde 3-phosphate dehydrogenase
GTT: glucose tolerance test
HFD: high fat diet
ICAM-1: intracellular cell adhesion molecule-1
IL: interleukin
iNOS: inducible nitric oxide synthase
IR: insulin receptor
IRS1: IR substrate 1
I.P.: intraperitoneally
LDLR^−/−^: low density lipoprotein receptor deficient mice
mTORC1: mammalian target of rapamycin complex 1
MCP-1: monocyte chemoattractant protein-1
PI3K: phosphoinositide 3-kinase
PKB: protein kinase B
PTP1B: protein tyrosine phosphatase 1B
TNFα: tumor necrosis factor α
VCAM-1: vascular cell adhesion molecule-1
VEGF: vascular endothelial growth factor
VEGFR: VEGF receptor
WHO: World Health Organization
