# Supplementary material for: Pharmacological inhibition of protein tyrosine phosphatase 1B protects against atherosclerotic plaque formation in the LDLR−/− mouse model of atherosclerosis
Source: Clin Sci (Lond). 2017 Sep 29;131(20):2489–501. doi: 10.1042/CS20171066 (PMC6365594; doi:10.1042/CS20171066)
Supplement: Supplementary file 1 [file cs-131-cs20171066_supp1.pdf]

Supplemental Figures

Pharmacological inhibition of protein tyrosine phosphatase 1B (PTP1B) protects against atherosclerotic plaque formation in the LDLR$^{-/-}$ mouse model of atherosclerosis.

D Thompson[1*], N Morrice[1], L Grant[1], S Le Sommer[1], EK Lees[1], N Mody[1], HM Wilson[1] & M Delibegovic[1*].

[1.] Institute of Medicine, Medical Sciences and Nutrition, University of Aberdeen, Aberdeen, UK

Supplemental Methods.

Food Intake

Food intake of all cohorts of mice was analysed during weeks 8 and 9 of the study.

Liver PTP1B Activity Assay

Liver from saline, single dose or chronically trodusquemine treated mice were homogenised in PTP1B lysis buffer (130mM NaCl, 20mM Tris-HCl (pH 7.5), 5mM EDTA, 1% Triton X-100, 0.5% NP-40, protease inhibitor cocktail) and normalised to 1mg of protein in 1ml of buffer. Subsequently 2µg of mouse PTP1B antibody was added and the samples incubated for 2hrs at 4°C with top-over-end mixing before being incubated for a further hr with protein A sepharose beads. Next, beads were washed twice in lysis buffer and twice in PTP1B activity buffer (100mM HEPES (pH 7.6), 2mM EDTA,150mM NaCl and 0.5mg/ml BSA) before being incubated at 30°C for 30 mins with shaking in activity buffer containing 1mM DTT and 200µM pp60c-Src C-terminal phospho-regulatory peptide (Enzo Life Sciences). Following incubation, 100µl on Biomol green reagent (Enzo Life Sciences) was added to 40µl of sample and after 30 mins incubation, the free phosphate was measured at 620nm.

Immunoblotting of ER Stress markers

Frozen aorta tissues were homogenised in 300µl of ice-cold Radioimmunoprecipitation assay (RIPA) buffer (10mM Tris-HCl pH 7.4, 150mM NaCl, 5mM EDTA pH 8.0, 1mM NaF, 0.1% SDS, 1% Triton X-100, 1% Sodium Deoxycholate with freshly added 1mM $NaVO_4$ and protease inhibitors) using a PowerGen 125 homogeniser and lysates normalised to 1µg per 1µl. Proteins were separated on a 4-12% Bis-Tris gel by SDS-PAGE and transferred onto nitrocellulose membrane. Membranes were probed for the following targets; p-eif2α (Ser51), total eif2α, BiP, pIRE1α (Ser 727), CHOP and GAPDH.

Supplemental Figure 1: Trodusquemine treatment reduces food intake. Food intake of HFD (**A**) and CHOW-fed (**B**) cohorts at week 9. PTP1B activity data of liver tissue from HFD-fed saline, single dose or chronically trodusquemine treated cohorts following terminal culls as assessed by inhibition of free phosphate production (C, n=4-5 per group). Data are represented as mean $\pm$ S.E.M. and analysed by unpaired two tailed t-tests where *p≤0.05 or ****p≤0.0001 when compared to saline control groups.

Supplemental Figure 2: Trodusquemine treatment does not improve ER stress.

(**A**) Western blot analysis of aortic tissues from saline, single dose or chronically treated HFD-fed mice injected with either saline or insulin immediately prior to culling. Quantification of p- pIRE1α (Ser 727) (**B**), BiP (**C**) p-eif2α (Ser 51) (**D**) and CHOP (**E**) represented in (**A)** Data are represented as mean $\pm$ S.E.M. and analysed by unpaired two-tailed t-tests.

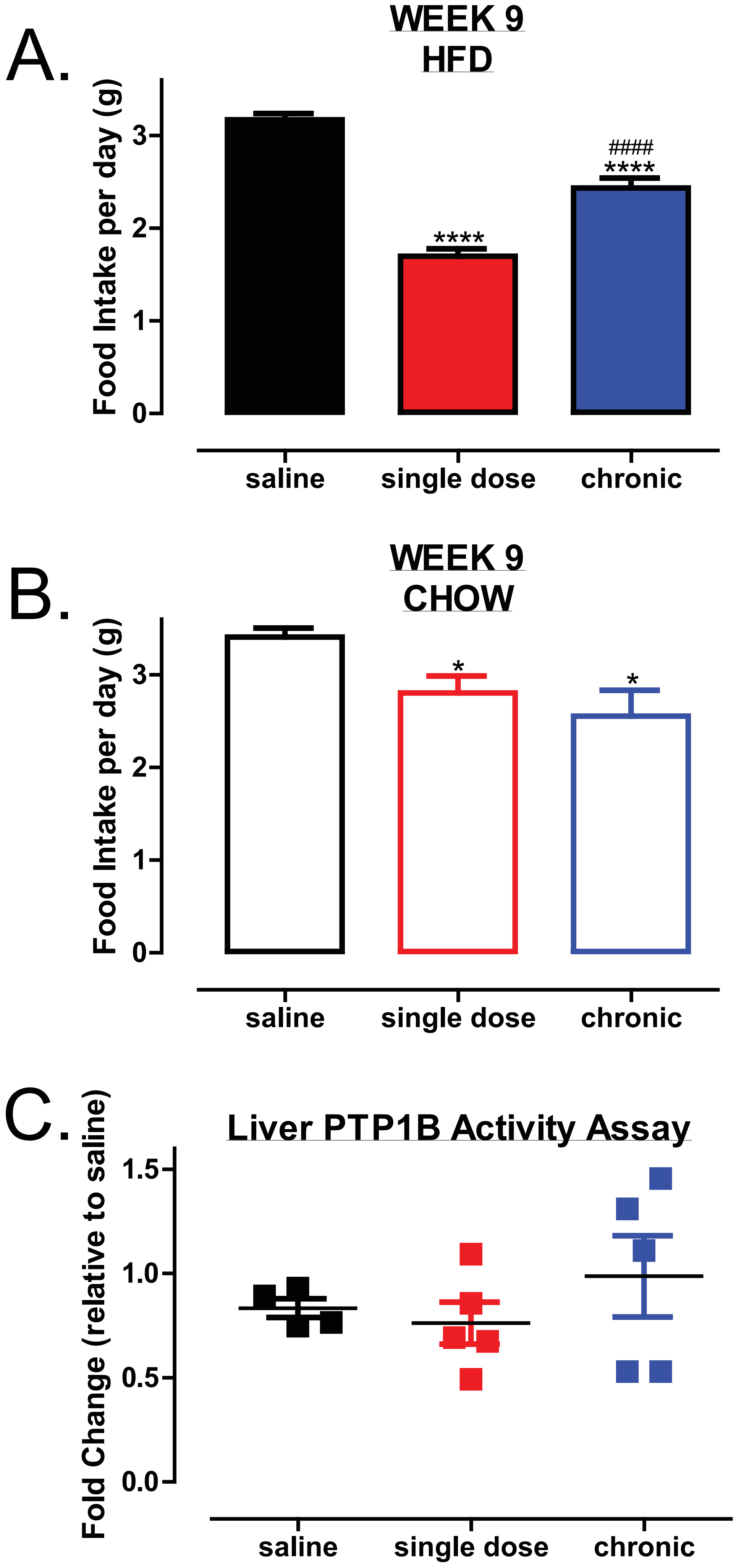

Supplemental Figure 1

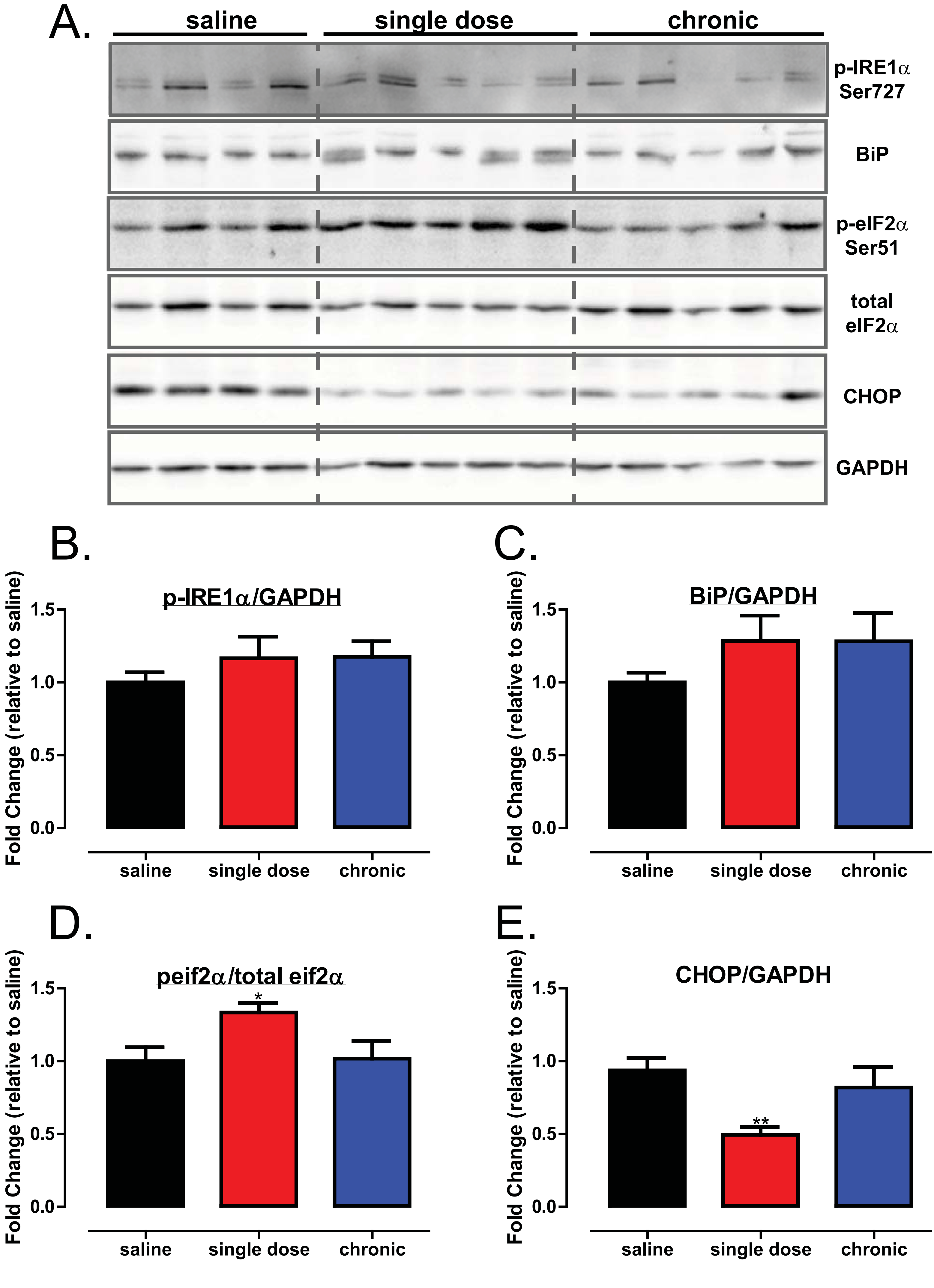

Supplemental Figure 2
